# Decreased circulating branched-chain amino acids are associated with development of Alzheimer’s disease in elderly individuals with mild cognitive impairment

**DOI:** 10.3389/fnut.2022.1040476

**Published:** 2022-12-14

**Authors:** Takeshi Ikeuchi, Mayuka Kanda, Hitomi Kitamura, Fumiyoshi Morikawa, Shuta Toru, Chika Nishimura, Kensaku Kasuga, Takayoshi Tokutake, Tetsuya Takahashi, Yasuko Kuroha, Nobuhiko Miyazawa, Shin Tanaka, Kumiko Utsumi, Kenjiro Ono, Satoshi Yano, Tadanori Hamano, Satoshi Naruse, Ryuji Yajima, Noriko Kawashima, Chikako Kaneko, Hisatsugu Tachibana, Yuki Yano, Yumiko Kato, Sakino Toue, Hiroko Jinzu, Akihiko Kitamura, Yuri Yokoyama, Eiji Kaneko, Minoru Yamakado, Kenji Nagao

**Affiliations:** ^1^Department of Molecular Genetics, Brain Research Institute, Niigata University, Niigata, Japan; ^2^Research Institute for Bioscience Products and Fine Chemicals, Ajinomoto Co., Inc., Kawasaki, Japan; ^3^Department of Psychiatry, Asahikawa Keisenkai Hospital, Asahikawa, Japan; ^4^Department of Neurology, Nitobe Memorial Nakano General Hospital, Tokyo, Japan; ^5^Kurumi Clinic, Tokyo, Japan; ^6^Department of Neurology, Brain Research Institute, Niigata University, Niigata, Japan; ^7^Department of Neurology, Nishiniigata Chuo Hospital, Niigata, Japan; ^8^Department of Neurosurgery, Kofu Neurosurgical Hospital, Kofu, Japan; ^9^Mishima Hospital, Niigata, Japan; ^10^Department of Psychiatry, Sunagawa City Medical Center, Sunagawa, Japan; ^11^Division of Neurology, Department of Medicine, Showa University School of Medicine, Tokyo, Japan; ^12^Faculty of Medical Sciences, Second Department of Internal Medicine, University of Fukui, Fukui, Japan; ^13^Department of Neurology, Midori Hospital, Niigata, Japan; ^14^Kawashima Neurology Clinic, Fujisawa, Japan; ^15^Southern TOHOKU Medical Clinic, Koriyama, Japan; ^16^Department of Neurology, Takatsuki General Hospital, Takatsuki, Japan; ^17^Institute for Innovation, Ajinomoto Co., Inc., Kawasaki, Japan; ^18^Research Team for Social Participation and Community Health, Tokyo Metropolitan Institute of Gerontology, Tokyo, Japan; ^19^Institute of Education, Tokyo Medical and Dental University, Tokyo, Japan; ^20^Department of Nursing, Ashikaga University, Ashikaga, Japan

**Keywords:** multicenter clinical study, protein malnutrition, biomarker discovery, MMSE–Mini-Mental State Examination, *APOE*

## Abstract

**Background:**

Nutritional epidemiology has shown that inadequate dietary protein intake is associated with poor brain function in the elderly population. The plasma free amino acid (PFAA) profile reflects nutritional status and may have the potential to predict future changes in cognitive function. Here, we report the results of a 2-year interim analysis of a 3-year longitudinal study following mild cognitive impairment (MCI) participants.

**Method:**

In a multicenter prospective cohort design, MCI participants were recruited, and fasting plasma samples were collected. Based on clinical assessment of cognitive function up to 2 years after blood collection, MCI participants were divided into two groups: remained with MCI or reverted to cognitively normal (“MCI-stable,” *N* = 87) and converted to Alzheimer’s disease (AD) (“AD-convert,” *N* = 68). The baseline PFAA profile was compared between the two groups. Stratified analysis based on apolipoprotein E ε4 (*APOE* ε4) allele possession was also conducted.

**Results:**

Plasma concentrations of all nine essential amino acids (EAAs) were lower in the AD-convert group. Among EAAs, three branched-chain amino acids (BCAAs), valine, leucine and isoleucine, and histidine (His) exhibited significant differences even in the logistic regression model adjusted for potential confounding factors such as age, sex, body mass index (BMI), and *APOE* ε4 possession (*p* < 0.05). In the stratified analysis, differences in plasma concentrations of these four EAAs were more pronounced in the *APOE* ε4-negative group.

**Conclusion:**

The PFAA profile, especially decreases in BCAAs and His, is associated with development of AD in MCI participants, and the difference was larger in the *APOE* ε4-negative population, suggesting that the PFAA profile is an independent risk indicator for AD development. Measuring the PFAA profile may have importance in assessing the risk of AD conversion in the MCI population, possibly reflecting nutritional status.

**Clinical trial registration:**

[https://center6.umin.ac.jp/cgi-open-bin/ctr/ctr_view.cgi?recptno=R000025322], identifier [UMIN000021965].

## Introduction

Alzheimer’s disease (AD) is a neurodegenerative disorder that occurs with high frequency in old age (1) and has significant social and economic consequences (2). For example, Japan is a superaging society, and the dementia population is estimated to grow from 4.4 million in 2018 to over 7 million by 2025, or one in five people aged 65 years or older (3). Caring for patients with cognitive decline requires the support of multiple parties, resulting in a significant social and economic burden (2). The accumulation of amyloid-β (Aβ) in the brain is hypothesized to play a causative, initiating role in AD. Although it is expected that some anti-amyloid agents will be approved as drugs, challenges in terms of coverage, price, and convenience are expected to remain. Even if the development of cognitive impairments could be delayed by pharmacotherapy, it is economically challenging to continue the treatment for such a long time after the preclinical phase.

From this perspective, a lifestyle modification approach is one of the realistic options to address dementia. When considered in relation to body mass index (BMI), low BMI is a clear risk factor for AD in elderly individuals. A recent study of 3,632 participants in the Framingham study reported that a lower BMI is associated with a higher risk of dementia in old age (>50 years) (4). In the Japanese population, it has also been reported that lower BMI is associated with a higher incidence of dementia (5). Inadequate dietary intake triggers low BMI and prefrailty/frailty, and the prefrailty/frailty leads to malnutrition, forming a vicious cycle of deteriorating health outcomes (6).

In this regard, changing dietary habits can be a feasible option. A long-term randomized controlled trial called the Finnish Geriatric Intervention Study to Prevent Cognitive Impairment and Disability (FINGER), a multidomain lifestyle-based intervention that includes dietary intervention to improve vascular and lifestyle-related risk factors, was found to maintain cognitive function and reduce the risk of cognitive decline in older adults at high risk of dementia (7). The World Health Organization (WHO) guidelines for cognitive decline and dementia risk reduction recommends a healthy, balanced diet for all adults (8). In particular, recent findings in nutritional epidemiological studies have shown that protein intake is particularly important for maintaining brain function in the elderly population (9–12). Recently, the quality of protein has also been studied, and a low amino acid score of breakfast has been associated with cognitive impairment (13).

Precision biomarkers linking nutritional status and cognitive decline have been intensively investigated (14) and several candidate markers based on cerebrospinal fluid (CSF) or blood samples were reported in both rodent model and clinical studies (15–18). We previously demonstrated the following as the first report of this clinical study (19). We cross-sectionally compared the concentrations of plasma free amino acid (PFAA) and albumin (Alb) in multicenter clinical study with 219 elderly individuals with MCI and 220 cognitively normal (CN) individuals. We found that the concentrations of essential amino acids (EAAs), such as Lys, His, and Thr, and Alb were lower in the MCI group (19). We are currently following up these individuals with MCI for up to 3 years to assess AD development and are developing a prognostic blood-based biomarker focusing on PFAAs. Here, we report an interim analysis mainly based on the 2-year follow-up status of 155 MCI individuals. Additionally, a stratified analysis based on apolipoprotein E ε4 (*APOE* ε4) allele possession is reported.

## Materials and methods

### Ethics statement

This study was carried out in accordance with the Declaration of Helsinki and approved by the ethics committee of respective institutions, including Niigata University and Ajinomoto Co., Inc. This study was registered at the University Hospital Medical Information Network Clinical Trials Registry under the number UMIN000021965. Written informed consent was obtained from all participants before participation in this study. All clinical information of the participants was anonymized prior to data analysis.

### Participants

**FIGURE 1:**
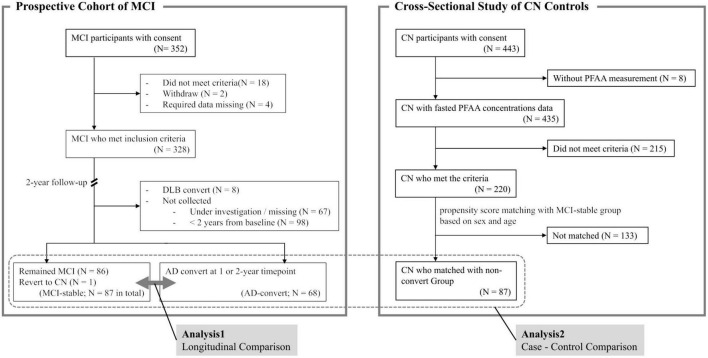
Summary of study design. MCI, mild cognitive impairment; AD, Alzheimer’s disease; DLB, dementia with Lewy bodies; CN, cognitively normal; PFAA, plasma free amino acid.

This is the interim analysis of a 3-year longitudinal study. Participants with MCI (*N* = 352) were recruited between September 2016 and December 2021 from the following 14 medical institutions: Niigata University Medical and Dental Hospital, Asahikawa Keisenkai Hospital, Nitobe Memorial Nakano General Hospital, Kurumi Clinic, Nishiniigata Chuo Hospital, Kofu Neurosurgical Hospital, Mishima Hospital, Sunagawa City Medical Center, Showa University Hospital, University of Fukui Hospital, Midori Hospital, Kawashima Neurology Clinic, Southern TOHOKU Medical Clinic, and Takatsuki General Hospital. Participants who had passed more than 2 years after inclusion were included in the current analysis (Figure 1).

Control participants (CN, *N* = 220) who received comprehensive health examinations were recruited from community-dwelling adults at four different cities across Japan (Hatoyama Town, Mitsuke City, Ashikaga City, and Kawasaki City) and from CN participants who visited Sunagawa City Medical Center (19).

### Cognitive evaluation and inclusion and exclusion criteria

Since this clinical study is a continuation of the previous report, the study is basically proceeding according to the protocol reported in the previous report (19). Neuropsychological assessments were conducted to evaluate cognitive function of the participants. The Mini-Mental State Examination (MMSE) was used to assess general cognitive function. The Wechsler Memory Scale-Revised Logical Memory II (WMS-R LM II) subtest or Clinical Dementia Rating (CDR) was used to assess memory function and the clinical severity of dementia. The Geriatric Depression Scale-15 (GDS-15) was used for the assessment of the degree of depression.

The following are the inclusion and exclusion criteria for the current study as previously described (19). The common criteria for MCI and CN participants were as follows: (1) aged 50 years or older; (2) not diagnosed as having dementia; (3) living independently; (4) able to undergo neuropsychological tests with an MMSE score of 24 points or higher; and (5) no depression and a GDS-15 score less than six points. In addition, the participant was included in the MCI group if either of the following was applicable: (1) WMS-R LM II score of 11 points or less if the participant had 16 years or more of education, nine points or less if the participant had 8 to 15 years of education, and six points or less if the participant had 7 years or less of education; and (2) a global CDR score of 0.5, which is comparable to the criteria used in the Japanese Alzheimer’s Disease Neuroimaging Initiative (J-ADNI) (20). For the CN participants who were recruited from community-dwelling older adults, the following criteria were additionally applied: (1) an MMSE score of 28–30 points or diagnosed as cognitively normal by a dementia specialist and (2) living independently. The exclusion criteria for MCI and CN were as follows: (1) the consumption of meals or any amino acid formulations, supplements, or beverages within 10 h before blood drawing; (2) less than 6 years of education; (3) current or previous treatment for alcohol addiction; (4) complications of cancer or liver cirrhosis; (5) dialysis treatment; (6) other neurodegenerative or psychiatric disorders; and (7) assessment as ineligible by medical doctors.

### Plasma free amino acid analysis

At baseline, 5 ml of venous blood were collected from antecubital veins under fasting conditions in the morning. The blood samples were collected into tubes containing EDTA 2Na as an anticoagulant and were immediately (<1 min) placed in ice water or an ice-cold cooling container (Forte Grow Medical Co., Ltd., Tochigi, Japan). PFAA concentrations were analyzed by high-performance liquid chromatography (HPLC)–electrospray ionization (ESI)–mass spectrometry (MS) by precolumn derivatization. The analytical methods in details have been described elsewhere (21, 22). Concentrations of the following 22 amino acids were determined: alanine (Ala), aminobutyric acid (α-ABA), arginine (Arg), asparagine (Asn), citrulline (Cit), glutamic acid (Glu), glutamine (Gln), glycine (Gly), histidine (His), isoleucine (Ile), leucine (Leu), lysine (Lys), methionine (Met), ornithine (Orn), phenylalanine (Phe), proline (Pro), serine (Ser), taurine (Tau), threonine (Thr), tryptophan (Trp), tyrosine (Tyr), and valine (Val).

### Blood biochemistry and apolipoprotein E genotype

The BMI of the participants was calculated based on their height and weight. The following blood parameters were measured in all participants: hemoglobin A1c (HbA1c), Alb, fasting blood glucose, and creatinine. In addition, the following blood variables were measured in all MCI participants: white blood cell (WBC) count, red blood cell (RBC) count, hemoglobin (Hb), hematocrit (Ht), platelet (PLT) count, insulin, total protein (TP), high-density lipoprotein cholesterol (HDLC), low-density lipoprotein cholesterol (LDLC), prealbumin, C-reactive protein (CRP), triglyceride (TG), blood urea nitrogen (BUN), uric acid (UA), calcium (Ca), iron (Fe), aspartate aminotransferase (AST), alanine aminotransferase (ALT), γ-glutamyl transpeptidase (γ-GTP), and folate. In all MCI participants, the following red blood cell indices were calculated based on RBC, Hb, and Ht measurements; mean corpuscular volume (MCV), mean corpuscular hemoglobin (MCH), and mean corpuscular hemoglobin concentration (MCHC).

Genomic DNAs were extracted from peripheral blood of all MCI participants using an automated DNA isolation system (QuickGene-Auto240L, Kurabo, Osaka, Japan). We determined *APOE* genotypes (rs429358 and rs7412) using TaqMan^®^ PCR Assays (Applied Biosystems, Foster City, CA, USA). In accordance with previous studies (23, 24), participants with one or two alleles of ε4 were defined as the *APOE* ε4-positive group and those without the ε4 allele as the *APOE* ε4-negative group.

### Follow-up

Follow-up of MCI participants was conducted at the recruiting hospital 1 and 2 years after blood collection and involved a clinical diagnosis of cognitive function, such as AD or MCI, by a medical doctor specializing in dementia. Participants who remained with MCI (*N* = 86) or revert to CN (*N* = 1) at the 2-year timepoint were categorized as the “MCI-stable” group collectively, and participants who developed AD at either the 1- or 2-year timepoint (*N* = 68) were categorized as the “AD-convert” group. Participants who did not return to the hospital at the 2-year timepoint were excluded from the current analysis. Participants who developed dementia types other than AD (such as dementia with Lewy bodies or vascular dementia) were also excluded.

### Statistical analysis

#### Baseline characteristics and plasma free amino acid profiles

To describe the distributions of baseline characteristics and PFAA profiles for both MCI-stable and AD-converted individuals, means and standard deviations or proportions are calculated. Welch’s *t*-test or Fisher’s exact test was applied to assess differences in baseline characteristics between the MCI-stable and AD-convert groups. Mono- or multivariable logistic regression was used to assess differences in baseline PFAA profiles. Multivariable models were used to adjust for potential cofounding factors such as age, sex, BMI, and *APOE* ε4 allele possession.

#### Cognitively normal control dataset preparation

To conduct comparison analysis between CN and MCI-stable or CN and AD-convert, CN individuals were selected using propensity score matching so that the number of matched CN was the same as that of MCI-stable participants (*N* = 87). Because it is known that the PFAA profile varies with age and sex (25), age and sex were used to calculate propensity scores.

#### Receiver operating characteristic curve analysis

To determine the capabilities of each PFAA to discriminate AD-converts, receiver operating characteristic (ROC) curve analysis was performed. In the comparison between the MCI-stable and AD-convert groups, the MCI-stable label was fixed as the control class label. On the other hand, in the comparison between CN and MCI-stable or CN and AD-convert, the CN label was fixed as a control class label. Therefore, an area under the ROC curve (AUC of ROC) value < 0.5 indicated that the amino acid concentration was lower in the case group than in the control group, whereas an AUC of ROC value > 0.5 indicated the reverse.

#### Stratified analysis by apolipoprotein E ε4 status

In the stratified analysis, the dataset of MCI individuals was split according to *APOE* ε4 positive/negative status. Mono- and multivariable logistic regression models were fit to each dataset to assess differences in baseline PFAA profiles. In multivariable analysis, age, sex, and BMI were used as cofounding factors.

#### Software

All statistical analyses were conducted using the R (ver. 4.0.3) software (R Foundation for Statistical Computing, Vienna, Austria).

## Results

### Baseline characteristics of the mild cognitive impairment-stable and Alzheimer’s disease-convert groups

Table 1 summarizes the baseline characteristics of MCI-stable and AD-convert individuals included in this study (MCI-stable; *N* = 87, AD-convert; *N* = 68). There were no obvious differences in age or sex between MCI-stable and AD-converted individuals. BMI was slightly lower in the AD-convert group, but the difference was not statistically significant. The MMSE score was significantly lower and the *APOE* ε4-positive ratio was significantly higher in the AD-convert group. Regarding biochemical tests, LDL-cholesterol was significantly higher and ALT and γ-GTP were significantly lower in the AD-convert group.

**TABLE 1:** Baseline characteristics of participants.

	MCI-stable	AD-convert	*P*-value
*N*	87	68	–
Age (year)	79.4 ± 5.7	80.3 ± 5.3	0.300
Sex (Male %)	Male; 30, Female; 57 (34.5%)	Male; 18, Female; 50 (26.5%)	0.300
BMI (kg/m^2^)	23.2 ± 3.4	22.3 ± 4.1	0.160
SBP (mmHg)	133.9 ± 18.7	133.8 ± 20.1	0.971
DBP (mmHg)	72.2 ± 13.7	71.1 ± 12.7	0.587
*APOE*ε4 (positive %)	negative; 65, positive; 22 (25.3%)	negative; 36, positive; 32 (47.1%)	**0.006**
MMSE	27.5 ± 2.0	25.4 ± 1.5	**<0.001**
GDS-15	1.7 ± 1.5	1.7 ± 1.3	0.914
Education (years) *N* (%)	≤9; 33 (37.9%)	≤9; 24 (35.3%)	0.895
	10–12; 35 (40.2%)	10–12; 27 (39.7%)	
	>12; 19 (21.8%)	>12; 17 (25.0%)	
HbA1c (%)	5.9 ± 0.7	5.9 ± 0.8	0.867
WBC (/μl)	5365.5 ± 1536.4	5113.2 ± 1562.3	0.317
RBC (×10^4^/μl)	420.2 ± 44.7	416.8 ± 45.7	0.649
Hb (g/dl)	13.2 ± 1.5	12.9 ± 1.3	0.272
Ht (%)	40.3 ± 4.0	39.6 ± 3.7	0.294
MCV (fl)	96.1 ± 5.2	95.3 ± 4.7	0.363
MCH (pg)	31.4 ± 2.1	31.1 ± 1.9	0.359
MCHC (%)	32.7 ± 0.9	32.6 ± 1.0	0.780
PLT (×10^4^/μl)	22.9 ± 5.3	23.4 ± 4.8	0.547
Insulin (μIl/ml)	7.8 ± 7.6	6.3 ± 5.5	0.172
TP (g/dl)	7.3 ± 0.4	7.3 ± 0.5	0.634
HDLC (mg/dl)	65.9 ± 17.6	66.8 ± 17.9	0.763
LDLC (mg/dl)	112.6 ± 23.9	126.0 ± 29.2	**0.003**
Alb (g/dl)	4.2 ± 0.3	4.2 ± 0.3	0.780
Prealbumin (mg/dl)	24.8 ± 4.5	24.0 ± 4.8	0.307
CRP (mg/dl)	0.2 ± 0.5	0.2 ± 0.5	0.837
Glucose (mg/dl)	110.9 ± 26.1	107.3 ± 33.4	0.473
TGs (mg/dl)	104.1 ± 55.7	95.4 ± 53.6	0.324
BUN (mg/dl)	16.8 ± 5.3	16.6 ± 4.4	0.778
Creatinine (mg/dl)	0.8 ± 0.2	0.7 ± 0.2	0.612
UA (mg/dl)	5.2 ± 1.3	4.8 ± 1.4	0.057
Ca (mg/dl)	9.2 ± 0.3	9.2 ± 0.4	0.881
Fe (μg/dl)	97.5 ± 34.3	92.5 ± 27.3	0.316
AST (U/L)	26.8 ± 16.4	24.4 ± 7.7	0.232
ALT (U/L)	21.6 ± 13.5	17.4 ± 7.7	**0.017**
γ-GTP (U/L)	38.0 ± 40.7	22.7 ± 13.3	**0.001**
Folate (ng/ml)	9.9 ± 4.4	10.1 ± 4.6	0.812

Continuous variables (age, BMI, blood pressure, MMSE, GDS-15, and blood test) are described as the mean ± SD. Fisher’s exact test was performed for categorical variables (sex, *APOE*ε4 allele, and education), and Welch’s *t*-test was performed for other variables. The following variables were partially missing (*N* of MCI-stable, *N* of AD-converted): BMI (5, 1), blood pressure (3, 0), CRP (5, 0), Ca (0, 1), and Fe (0, 1). BMI, body mass index; SBP, systolic blood pressure; DBP, diastolic blood pressure; MMSE, Mini-Mental State Examination; GDS-15, Geriatric depression scale-15; HbA1c, hemoglobin A1c; WBC, white blood cell count; RBC, red blood cell count; Hb, hemoglobin; Ht, hematocrit; MCV, mean corpuscular volume; MCH, mean corpuscular hemoglobin; MCHC, mean corpuscular hemoglobin concentration; PLT, platelet count; TP, total protein; HDLC, high-density lipoprotein cholesterol; LDLC, low-density lipoprotein cholesterol; Alb, albumin; CRP, C-reactive protein; TG, triglyceride; BUN, blood urea nitrogen; UA, uric acid; Ca, calcium; Fe, iron; AST, aspartate aminotransferase; ALT, alanine transaminase; γ-GTP, γ-glutamyl transpeptidase. Bold text indicates statistical significance based on a *p*-value less than 0.05.

### Baseline plasma free amino acid profiles of mild cognitive impairment-stable and Alzheimer’s disease-convert groups

**FIGURE 2:**
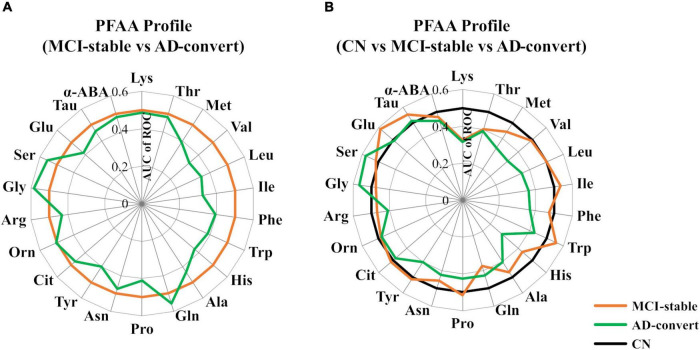
Comparison of plasma free amino acid (PFAA) concentrations among mild cognitive impairment (MCI)-stable, Alzheimer’s disease (AD)-convert, and cognitively normal (CN) individuals. Differences in PFAA concentrations between groups are described by the receiver operating characteristic (ROC) of an area under the curve (AUC). **(A)** The MCI-stable group was set as the control. **(B)** The CN group was set as the control. In the CN group, taurine data were partially missing (*N* = 48 were missing).

Table 2 shows the baseline concentrations of 22 PFAAs of MCI-stable and AD-converted individuals. Concentrations of all nine EAAs were lower in AD-converted individuals. In particular, the concentrations of three branched-chain amino acids (BCAAs), Val, Leu, and Ile, were significantly lower in the AD-convert group even in the model adjusted for potential confounding factors. The concentration of His was also significantly lower in the AD-converted group. Regarding non-essential amino acids (NEAAs), Ser, Gly, and Gln were slightly higher in AD-converted individuals but were not significant in any statistical models. The results were similar when case group was expanded to all cause dementia (*N* = 76) (Supplementary Tables 1, 2). Additionally, we applied ROC curve analysis for 22 PFAAs (Figure 2A and Supplementary Figure 1).

**TABLE 2:** Comparison of plasma free amino acid (PFAA) concentrations between mild cognitive impairment (MCI)-stable and Alzheimer’s disease (AD)-convert.

Amino acid (μM)	MCI-stable (*N* = 87)	AD-convert (*N* = 68)	*P*-value	
			
	Mean ± SD	Mean ± SD	Crude model	Adjusted model
Lys	178.1 ± 29.3	177.0 ± 28.5	0.805	0.802
Thr	110.6 ± 21.0	109.7 ± 23.8	0.802	0.810
Met	24.2 ± 4.9	22.6 ± 4.1	**0.028**	0.055
Val	210.9 ± 38.4	190.3 ± 40.8	**0.002**	**0.014**
Leu	111.0 ± 22.3	99.5 ± 23.8	**0.003**	**0.023**
Ile	60.1 ± 13.4	53.3 ± 14.8	**0.005**	**0.016**
Phe	61.3 ± 8.5	59.8 ± 14.9	0.439	0.551
Trp	51.8 ± 9.0	48.2 ± 10.3	**0.025**	0.084
His	77.5 ± 10.7	73.3 ± 6.9	**0.008**	**0.049**
Ala	352.7 ± 87.1	333.2 ± 84.6	0.164	0.154
Gln	584.7 ± 65.2	595.1 ± 74.2	0.351	0.390
Pro	152.2 ± 58.5	135.0 ± 52.3	0.065	0.129
Asn	45.3 ± 7.4	44.5 ± 6.3	0.443	0.526
Tyr	65.4 ± 13.1	61.8 ± 14.7	0.115	0.175
Cit	38.2 ± 8.8	37.6 ± 10.0	0.708	0.391
Orn	58.3 ± 13.3	61.9 ± 21.9	0.213	0.416
Arg	93.1 ± 18.0	89.6 ± 18.1	0.230	0.060
Gly	216.5 ± 55.7	231.7 ± 60.1	0.108	0.125
Ser	106.6 ± 22.0	110.6 ± 16.8	0.223	0.243
Glu	36.7 ± 20.5	30.9 ± 17.3	0.066	0.224
Tau	57.9 ± 40.7	51.3 ± 11.5	0.259	0.251
α-ABA	17.4 ± 5.1	17.3 ± 5.0	0.975	0.721

All data are described as the mean ± SD. Crude model: logistic regression with no covariates. Adjusted model: logistic regression adjusted for age, sex, BMI, and *APOE* ε4 possession.

Bold text indicates statistical significance according to a *p*-value less than 0.05.

### Baseline plasma free amino acid profiles of cognitively normal and mild cognitive impairment who developed/did not develop Alzheimer’s disease

We also applied ROC curve analysis setting CN participants as the control group (Figure 2B and Supplementary Table 3). Interestingly, in the comparison between CN and AD-converted individuals, the concentrations of plasma Met, Val, Leu, and His were lower in the AD-convert group, whereas in the comparison between CN and MCI-stable individuals, these amino acids did not show notable differences. In contrast, the plasma concentration of Lys was lower in both the AD-converted and MCI-stable groups than in the CN group. Plasma Gln was significantly lower and Glu was significantly higher only in the MCI-stable group.

### Stratified analysis by apolipoprotein E ε4 positive/negative status

**FIGURE 3:**
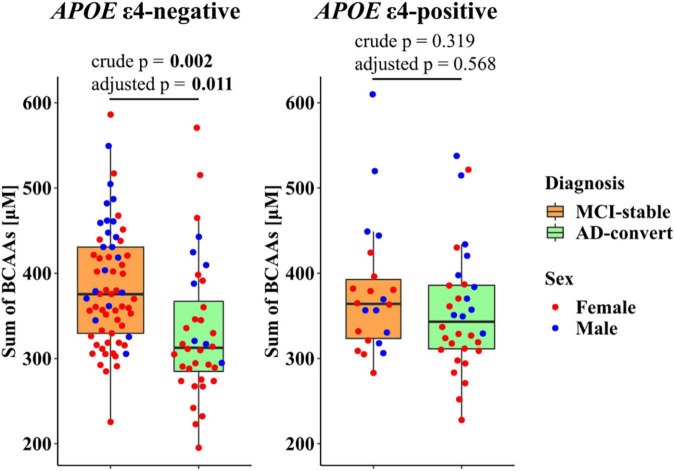
Stratified analysis of plasma branched-chain amino acid (BCAA) concentrations in mild cognitive impairment (MCI) individuals by apolipoprotein E ε4 (*APOE*ε4) positive/negative status. Red and blue dots represent females and males, respectively. Crude *p*: *p*-value calculated based on the logistic regression model with no covariates. Adjusted *p*: *p*-value calculated based on the logistic regression model adjusted for age, sex, and BMI. Bold text indicates statistical significance according to a *p*-value less than 0.05.

To further investigate the relationship of genetic risk factors with the PFAA profile, we conducted a comparison between the MCI-stable and AD-converted groups, stratifying by *APOE* ε4 positive/negative status. Because the plasma concentrations of the three BCAAs were highly correlated with each other (*r* > 0.87), the sum of the three BCAA concentrations was used as a representative indicator. In the *APOE* ε4-negative group, difference in the sum of three BCAA concentrations between the MCI-stable and AD-converted groups was statistically significant, whereas it was not significant in the *APOE* ε4-positive group (Figure 3). Similarly, differences in three individual BCAAs, His, Gly, and Glu between the MCI-stable and AD-converted groups were greater in the *APOE* ε4-negative group than in the *APOE* ε4-positive group (Supplementary Table 4 and Supplementary Figures 2, 3).

## Discussion

In the current analysis, we investigated the association of AD development within 2 years and the baseline PFAA profile of MCI participants. The AD-converted group showed lower concentrations of EAAs, especially three BCAAs: Val, Leu, and Ile. Additionally, in the stratified analysis, the association of plasma BCAA concentrations and AD development was more pronounced in the *APOE* ε4-negative group.

Recent findings in nutritional epidemiology have shown that protein intake is important for maintaining brain function in the elderly. For example, the amount of protein intake of dementia patients is significantly lower compared with healthy elderly individuals (9, 10, 26). The protein intake in the elderly is positively associated with memory function (27, 28). Elderly individuals with higher levels of protein intake have a lower risk of MCI (11), and lower levels of Aβ accumulation in the brain (12). These reports provide some insight into the relationship between protein intake and the risk of developing dementia and AD. In a previous study, we reported that MCI individuals showed lower plasma EAA concentrations than CN individuals based on case-control design analysis, demonstrating that among EAAs, Lys, Thr, and Met showed the largest differences (19). In contrast, the current longitudinal study revealed that plasma BCAA concentrations showed the greatest difference between the MCI-stable and AD-converted groups among the MCI population. It is known that some individuals with MCI convert to AD, while others do not, and it is intriguing that these differences are reflected in the differences in plasma BCAA and His concentrations. It is worth noting that in the current study, the differences in serum concentrations of Alb and prealbumin, which are traditionally used indicators of nutritional status, were not statistically significant between the MCI-stable and AD-converted groups (Table 1). Recent position paper by American Society for Parenteral and Enteral Nutrition (ASPEN) pointed out that serum Alb and prealbumin do not serve as valid proxy measures of total body protein or total muscle mass and they must be recognized as inflammatory markers associated with “nutrition risk” (29). Rather, our study indicates that PFAA may be suitable indicator in this context. Interestingly, one study showed that low concentration of serum Val was associated with both brain atrophy and cognitive decline, but not CSF Aβ_1–42_ level nor CSF Aβ_1–42_/total tau ratio (30). The concentration of BCAAs in the blood might reflect AD pathology other than Aβ or tau accumulation, such as inflammation or oxidative stress in the brain. Compared to Lys and Thr, which are commonly decreased in MCI compared to CN participants, circulating BCAAs and His may be related to pathological conditions and/or nutritional status, and future detailed studies are warranted.

Recently, multiple reports have emerged on the relationship between changes in the PFAA profile and dementia. An NMR-based metabolomics study based on eight prospective cohorts with 22,623 participants showed that lower serum BCAA concentrations were associated with an increased risk of all types of dementia and AD (31). Recently, a study based on circulating plasma metabolomic data of 110,655 participants in the UK Biobank reported similar results regarding the decrease of plasma BCAAs and the development of dementia (32). It should be noted that in our study, a similar association was found even in the East Asian population. Eating habits and body composition vary across countries, which makes it difficult to compare epidemiological findings between different cultural areas. For example, the average meat consumption per person in Japan is less than half of that in the US (33), and the average BMI and obesity rate of Japanese people are the lowest among OECD member countries (34). Additionally, higher milk consumption was associated with a decrease in executive function or global cognition in a US cohort study (35), whereas higher dairy and milk consumption was associated with a lower risk of all-cause dementia or vascular dementia in two independent Japanese cohort studies (36, 37). Nonetheless, a similar association between the PFAA profile and AD development among diverse food cultures suggests that plasma BCAA concentrations have universal importance in this context.

In this study, differences in *APOE* ε4 possession showed different strengths of association. The Rotterdam Study, which is a previous study on the relationship between lifestyle and the *APOE* allele, demonstrated that a healthy lifestyle including a healthy diet reduced the risk of dementia in individuals without the *APOE* ε4 allele (38). This suggests that a healthy lifestyle, including a balanced diet, is an independent protective factor against dementia, and *APOE* ε4-negative individuals are more sensitive to lifestyle modification, which is in accordance with our findings that *APOE* ε4-negative MCI individuals had a stronger association between the PFAA profile and AD development. However, the importance of EAAs in *APOE* ε4*-*positive individuals needs future investigation because in our stratified analysis, plasma EAA concentrations of the AD-converted group were lower even in the *APOE* ε4*-*positive population, but the effect size might be smaller than that in the *APOE* ε4*-*negative population.

Precision biomarkers are key to the preventions tailored to an individual’s biological and nutritional status (14). When considering nutritional interventions based on the current results, the molecular mechanism that links circulating EAAs and development of dementia is important. As a lower plasma BCAA concentration is associated with the progression of sarcopenia (39, 40) one reason may be the loss of muscle strength and the associated decrease in the amount of activity. A decline in handgrip strength has been demonstrated to be an important indicator of dementia (41). Additionally, daily exercise has been reported to help prevent dementia (42). A higher non-grain-derived BCAA intake was associated with handgrip strength among Korean older adults (43), and nutritional intake that supports daily exercise might be desirable. Another perspective is that low-protein diets have been reported to decrease essential amino acid influx to the brain (44) and cause inflammation in the brain, which may be one of the contributing factors of dementia (45, 46). In a previous study using tauopathy model mice, a decrease in circulating EAAs triggered inflammation in the brain. EAA ingestion reversed this effect by competitively inhibiting kynurenine, an initiator of a pathway inducing neuroinflammatory gliosis and neurotoxicity in the brain (45). A recent randomized clinical trial also suggested dietary EAA supplementation improved cognitive function in middle-aged and older adults (47). Another group reported that supplementation with a BCAA mixture in senior malnourished patients improved both MMSE scores and mitochondrial function in peripheral blood mononuclear cells (48). In summary, maintaining an adequate concentration of circulating amino acids by a balanced diet may be protective against cognitive decline potentially by (1) maintaining muscle mass, (2) preventing brain inflammation, and (3) improving mitochondrial function. Future intervention studies on these populations are needed.

This study has several limitations. First, our study did not include molecular imaging or CSF biomarkers, so we were unable to stratify participants with MCI or CN based on the underlying molecular pathology. Second, the study design is based on observational research, and there could be reverse causality. Progressing MCI individuals might have poorer appetite and/or more abnormal eating habits compared to stable MCI individuals, which might have reflected to the imbalance of PFAA profile. Third, factors that may affect PFAAs and cognitive function, such as diet, muscle mass, and physical activity were not included in the current analysis. In future studies, it will be particularly important to examine the correlation with protein nutritional status. Follow-up of the participants through 3 years is currently ongoing, and the relationship with the conversion to AD will be analyzed with other indicators that we were unable to analyze this time in future analyses.

This interim analysis of a multicenter longitudinal study revealed a significant relationship with development of AD and the PFAA profile, especially a decrease in circulating BCAAs and His among MCI participants. In the stratified analysis by *APOE* ε4 allele positive/negative status, the difference in the PFAA profile with or without AD development was more pronounced in the *APOE* ε4-negative group, suggesting that the PFAA profile is an independent risk indicator of genetic factors. Further studies are necessary to clarify the relationship between the PFAA profile and lifestyle factors, including nutritional intake.

## Data availability statement

The original contributions presented in the study are included in the article/Supplementary material, further inquiries can be directed to the corresponding authors.

## Ethics statement

The studies involving human participants were reviewed and approved by the Ethics Committee on Genetic Analysis of Niigata University and Ethics Committee of Ajinomoto Co., Inc. The patients/participants provided their written informed consent to participate in this study.

## Author contributions

TI and KN: conceptualization and funding acquisition. MK: data curation, formal analysis, software, and visualization. FM, STor, CN, KK, TTo, TTa, YKu, NM, STan, KU, KO, SY, TH, SN, RY, NK, CK, HT, AK, YYo, EK, MY, HK, YYa, HJ, and MK: investigation. HK, YYa, MK, YKa, and KN: methodology. TI, HK, and STou: project administration. MK, HK, YKa, and KN: resources. KN: supervision. MK and KN: validation. TI, HK, YYa, MK, and KN: writing—original draft. TI, MK, and KN: writing—review and editing. All authors have read and agreed to the published version of the manuscript.

## Glossary

AD: Alzheimer’s disease
Ala: alanine
Alb: albumin
ALT: alanine transaminase
*APOE*: apolipoprotein E
*APOE* ε4: apolipoprotein E ε4
Arg: arginine
Asn: asparagine
ASPEN: American Society for Parenteral and Enteral Nutrition
AST: aspartate aminotransferase
AUC: an area under the curve
Aβ: amyloid-β
BCAA: branched-chain amino acid
BMI: body mass index
BUN: blood urea nitrogen
Ca: calcium
CDR: Clinical Dementia Rating
Cit: citrulline
CN: cognitively normal
CRP: C-reactive protein
CSF: cerebrospinal fluid
DBP: diastolic blood pressure
DLB: dementia with Lewy bodies
EAA: essential amino acid
ESI: electrospray ionization
Fe: iron
GDS-15: Geriatric Depression Scale-15
Gln: glutamine
Glu: glutamic acid
Gly: glycine
Hb: hemoglobin
HbA1c: hemoglobin A1c
HDLC: high-density lipoprotein cholesterol
His: histidine
HPLC: high-performance liquid chromatography
Ht: hematocrit
Ile: isoleucine
LDLC: low-density lipoprotein cholesterol
Leu: leucine
Lys: lysine
MCH: mean corpuscular hemoglobin
MCHC: mean corpuscular hemoglobin concentration
MCI: mild cognitive impairment
MCV: mean corpuscular volume
Met: methionine
MMSE: Mini-Mental State Examination
MS: mass spectrometry
NEAA: non-essential amino acid
Orn: ornithine
PFAA: plasma free amino acid
Phe: phenylalanine
PLT: platelet
Pro: proline
RBC: red blood cell
ROC: receiver operating characteristic
SBP: systolic blood pressure
Ser: serine
Tau: taurine
TG: triglyceride
Thr: threonine
TP: total protein
Trp: tryptophan
Tyr: tyrosine
UA: uric acid
Val: valine
WBC: white blood cell
WMS-R: Wechsler Memory Scale-Revised
WMS-R LM II: Wechsler Memory Scale-Revised Logical Memory II
α-ABA: aminobutyric acid
γ-GTP: γ-glutamyl transpeptidase.
