# Supplementary material for: Decreased circulating branched-chain amino acids are associated with development of Alzheimer’s disease in elderly individuals with mild cognitive impairment
Source: Front Nutr. 2022 Dec 14;9:1040476. doi: 10.3389/fnut.2022.1040476 (PMC9794986; doi:10.3389/fnut.2022.1040476)
Supplement: Supplementary file 1 [file Data_Sheet_1.docx]

Supplementary Material

# **Supplementary Table 1: Baseline characteristics of DLB (dementia with Lewy bodies)-converted participants.**

Continuous variables (age, BMI, blood pressure, MMSE, GDS-15) are described as the mean ± SD.

|  | DLB-convert |
| --- | --- |
| N | 8 |
| Age [year] | 81.4 ± 6.4 |
| Sex | Male; 6, Female; 2 |
| (Male %) | (75.0%) |
| BMI [kg/m^2^] | 23.0 ± 2.3 |
| SBP [mmHg] | 133.0 ± 26.3 |
| DBP [mmHg] | 75.8 ± 15.2 |
| *APOE* ε4  (positive %) | negative; 3, positive; 5 |
|  | (62.5%) |
| MMSE | 26.8 ± 2.3 |
| GDS-15 | 1.6 ± 1.5 |
| Education [years] | ≦9; 3 (37.5%) |
| N (%) | 10-12; 3 (37.5%) |
|  | >12; 2 (25.0%) |

# **Supplementary Table 2. Comparison of PFAA concentrations between MCI-stable and all cause dementia-converted individuals.**

The all cause dementia-converted individuals consist of AD-convert (N = 68) and DLB-convert (N = 8). All data are described as the mean ± SD. Crude model: logistic regression with no covariates. Adjusted model: logistic regression adjusted for age, sex, BMI and *APOE* ε4 possession. Bold text indicates statistical significance according to a p value less than 0.05.

| Amino Acid  [µM] | MCI-stable  (N = 87) | all cause  dementia-converted  (N = 76) | p value | |
| --- | --- | --- | --- | --- |
|  | Mean ± SD | Mean ± SD | crude model | adjusted model |
| Lys | 178.1 ± 29.3 | 178.2 ± 28.2 | 0.984 | 0.692 |
| Thr | 110.6 ± 21.0 | 110.5 ± 23.3 | 0.991 | 0.946 |
| Met | 24.2 ± 4.9 | 22.7 ± 4.1 | **0.031** | **0.032** |
| Val | 210.9 ± 38.4 | 192.6 ± 40.5 | **0.005** | **0.012** |
| Leu | 111.0 ± 22.3 | 100.6 ± 23.2 | **0.005** | **0.015** |
| Ile | 60.1 ± 13.4 | 54.1 ± 14.7 | **0.010** | **0.012** |
| Phe | 61.3 ± 8.5 | 59.6 ± 14.3 | 0.371 | 0.400 |
| Trp | 51.8 ± 9.0 | 48.4 ± 10.0 | **0.026** | 0.067 |
| His | 77.5 ± 10.7 | 73.6 ± 7.1 | **0.010** | **0.041** |
| Ala | 352.7 ± 87.1 | 333.9 ± 85.4 | 0.168 | 0.115 |
| Gln | 584.7 ± 65.2 | 600.9 ± 75.0 | 0.144 | 0.203 |
| Pro | 152.2 ± 58.5 | 137.3 ± 52.2 | 0.095 | 0.141 |
| Asn | 45.3 ± 7.4 | 44.6 ± 6.2 | 0.483 | 0.438 |
| Tyr | 65.4 ± 13.1 | 61.6 ± 14.1 | 0.083 | 0.101 |
| Cit | 38.2 ± 8.8 | 38.6 ± 11.1 | 0.793 | 0.693 |
| Orn | 58.3 ± 13.3 | 62.0 ± 21.0 | 0.176 | 0.421 |
| Arg | 93.1 ± 18.0 | 91.7 ± 18.6 | 0.622 | 0.169 |
| Gly | 216.5 ± 55.7 | 232.5 ± 58.4 | 0.079 | 0.070 |
| Ser | 106.6 ± 22.0 | 110.1 ± 16.5 | 0.257 | 0.225 |
| Glu | 36.7 ± 20.5 | 29.7 ± 16.9 | **0.023** | 0.098 |
| Tau | 57.9 ± 40.7 | 50.9 ± 11.1 | 0.214 | 0.199 |
| α-ABA | 17.4 ± 5.1 | 17.0 ± 4.9 | 0.682 | 0.999 |

# **Supplementary Table 3: Characteristics of cognitively normal (CN) participants.**

Continuous variables (age, BMI, MMSE, blood test and plasma amino acid concentration) are described as the mean ± SD. Dunnett’s test was performed, and the CN group was set as the control group. Bold text indicates statistical significance according to a p value less than 0.05.

|  | CN (N = 87) |
| --- | --- |
| Age [year] | 79.6 ± 5.8 |
| Sex  (Male%) | Male; 35, Female; 52  (40.2%) |
| BMI [kg/m^2^] | 22.5 ± 2.8 |
| MMSE | 29.3 ± 0.8 |
| HbA1c [%] | 5.8 ± 0.5 |
| Alb [g/dL] | 4.4 ± 0.3 |
| Glucose [mg/dL] | 105.0 ± 20.6 |
| Creatinine [mg/dL] | 0.8 ± 0.2 |

| Amino Acid  [µM] | Mean ± SD | p value  (vs. MCI-stable) | p value  (vs. AD-convert) |
| --- | --- | --- | --- |
| Lys | 194.0 ± 29.4 | **< 0.001** | **< 0.001** |
| Thr | 117.8 ± 21.9 | 0.053 | 0.059 |
| Met | 24.8 ± 4.2 | 0.602 | **0.002** |
| Val | 209.6 ± 32.3 | 0.959 | **0.003** |
| Leu | 109.6 ± 20.4 | 0.870 | **0.012** |
| Ile | 58.0 ± 12.4 | 0.479 | 0.072 |
| Phe | 62.1 ± 8.7 | 0.749 | 0.427 |
| Trp | 50.3 ± 8.8 | 0.457 | 0.304 |
| His | 79.4 ± 9.1 | 0.340 | **< 0.001** |
| Ala | 359.9 ± 80.8 | 0.800 | 0.090 |
| Gln | 610.4 ± 59.4 | **0.014** | 0.297 |
| Pro | 144.6 ± 47.5 | 0.555 | 0.401 |
| Asn | 46.7 ± 7.7 | 0.382 | 0.089 |
| Tyr | 64.1 ± 11.1 | 0.707 | 0.484 |
| Cit | 38.2 ± 10.2 | 1.000 | 0.926 |
| Orn | 60.5 ± 19.7 | 0.597 | 0.883 |
| Arg | 94.7 ± 19.7 | 0.800 | 0.167 |
| Gly | 215.9 ± 45.9 | 0.996 | 0.139 |
| Ser | 105.8 ± 18.2 | 0.951 | 0.169 |
| Glu | 29.6 ± 12.1 | **0.012** | 0.845 |
| α-­ABA | 17.8 ± 4.1 | 0.770 | 0.777 |

# **Supplementary Table 4. Stratified analysis of MCI individuals by *APOE4* positive/negative status.**

crude model: logistic regression with no covariates. Adjusted model: logistic regression adjusted for age, sex and BMI. Bold text indicates statistical significance according to a p value less than 0.05.

| Amino Acid [µM] | *APOE4*-negative group | | | | *APOE4*-positive group | | | |
| --- | --- | --- | --- | --- | --- | --- | --- | --- |
|  | MCI-stable (N = 65) | AD-convert (N = 36) | p value (crude) | p value (adjusted) | MCI-stable (N = 22) | AD-convert (N = 32) | p value (crude) | p value (adjusted) |
|  | Mean ± SD | Mean ± SD |  |  | Mean ± SD | Mean ± SD |  |  |
| Lys | 178.9 ± 29.0 | 172.9 ± 30.4 | 0.330 | 0.995 | 175.8 ± 30.6 | 181.5 ± 25.9 | 0.458 | 0.443 |
| Thr | 109.9 ± 20.9 | 111.0 ± 24.9 | 0.817 | 0.648 | 112.5 ± 21.6 | 108.2 ± 22.9 | 0.486 | 0.463 |
| Met | 24.0 ± 4.5 | 22.6 ± 4.0 | 0.122 | 0.245 | 24.8 ± 5.9 | 22.5 ± 4.3 | 0.115 | 0.174 |
| Val | 211.9 ± 38.3 | 184.7 ± 42.3 | **0.003** | **0.013** | 208.0 ± 39.4 | 196.6 ± 38.8 | 0.292 | 0.495 |
| Leu | 111.4 ± 21.2 | 94.8 ± 24.3 | **0.001** | **0.010** | 110.0 ± 25.9 | 104.8 ± 22.4 | 0.432 | 0.835 |
| Ile | 60.3 ± 13.4 | 51.6 ± 15.2 | **0.006** | **0.020** | 59.3 ± 13.7 | 55.3 ± 14.2 | 0.304 | 0.451 |
| Phe | 61.3 ± 8.3 | 60.4 ± 18.2 | 0.741 | 0.624 | 61.2 ± 9.2 | 59.1 ± 10.2 | 0.429 | 0.613 |
| Trp | 51.8 ± 8.5 | 47.6 ± 12.2 | 0.051 | 0.179 | 51.8 ± 10.5 | 48.9 ± 7.8 | 0.250 | 0.334 |
| His | 78.0 ± 11.5 | 72.0 ± 7.1 | **0.009** | **0.044** | 76.2 ± 7.8 | 74.8 ± 6.5 | 0.474 | 0.893 |
| Ala | 355.9 ± 86.0 | 318.1 ± 80.5 | **0.036** | 0.087 | 343.1 ± 91.6 | 350.2 ± 87.1 | 0.769 | 0.860 |
| Gln | 586.0 ± 70.3 | 591.0 ± 84.1 | 0.744 | 0.828 | 580.9 ± 48.3 | 599.8 ± 62.1 | 0.236 | 0.171 |
| Pro | 158.0 ± 63.5 | 136.8 ± 57.3 | 0.107 | 0.148 | 135.1 ± 36.2 | 133.0 ± 46.8 | 0.858 | 0.840 |
| Asn | 45.5 ± 7.8 | 43.3 ± 6.0 | 0.145 | 0.186 | 44.9 ± 6.1 | 45.8 ± 6.4 | 0.581 | 0.343 |
| Tyr | 66.0 ± 12.5 | 62.0 ± 16.5 | 0.169 | 0.221 | 63.6 ± 15.0 | 61.7 ± 12.6 | 0.598 | 0.590 |
| Cit | 38.2 ± 8.8 | 37.6 ± 8.9 | 0.768 | 0.405 | 38.2 ± 8.9 | 37.6 ± 11.4 | 0.830 | 0.954 |
| Orn | 57.5 ± 13.4 | 59.8 ± 20.4 | 0.487 | 0.603 | 60.7 ± 13.3 | 64.2 ± 23.6 | 0.525 | 0.566 |
| Arg | 92.0 ± 17.4 | 89.4 ± 18.3 | 0.477 | 0.365 | 96.3 ± 19.6 | 89.8 ± 18.2 | 0.213 | 0.144 |
| Gly | 217.3 ± 58.8 | 246.2 ± 63.2 | **0.028** | **0.034** | 214.2 ± 46.8 | 215.4 ± 52.8 | 0.930 | 0.804 |
| Ser | 105.8 ± 22.6 | 113.7 ± 18.9 | 0.081 | 0.118 | 109.0 ± 20.5 | 107.0 ± 13.5 | 0.655 | 0.387 |
| Glu | 39.1 ± 21.3 | 28.1 ± 13.7 | **0.010** | **0.049** | 29.8 ± 16.7 | 34.0 ± 20.4 | 0.420 | 0.478 |
| Tau | 54.2 ± 22.5 | 51.0 ± 12.3 | 0.452 | 0.473 | 69.0 ± 71.2 | 51.5 ± 10.8 | 0.382 | 0.436 |
| α-ABA | 17.4 ± 5.1 | 16.7 ± 5.5 | 0.495 | 0.887 | 17.1 ± 5.1 | 18.0 ± 4.4 | 0.477 | 0.456 |


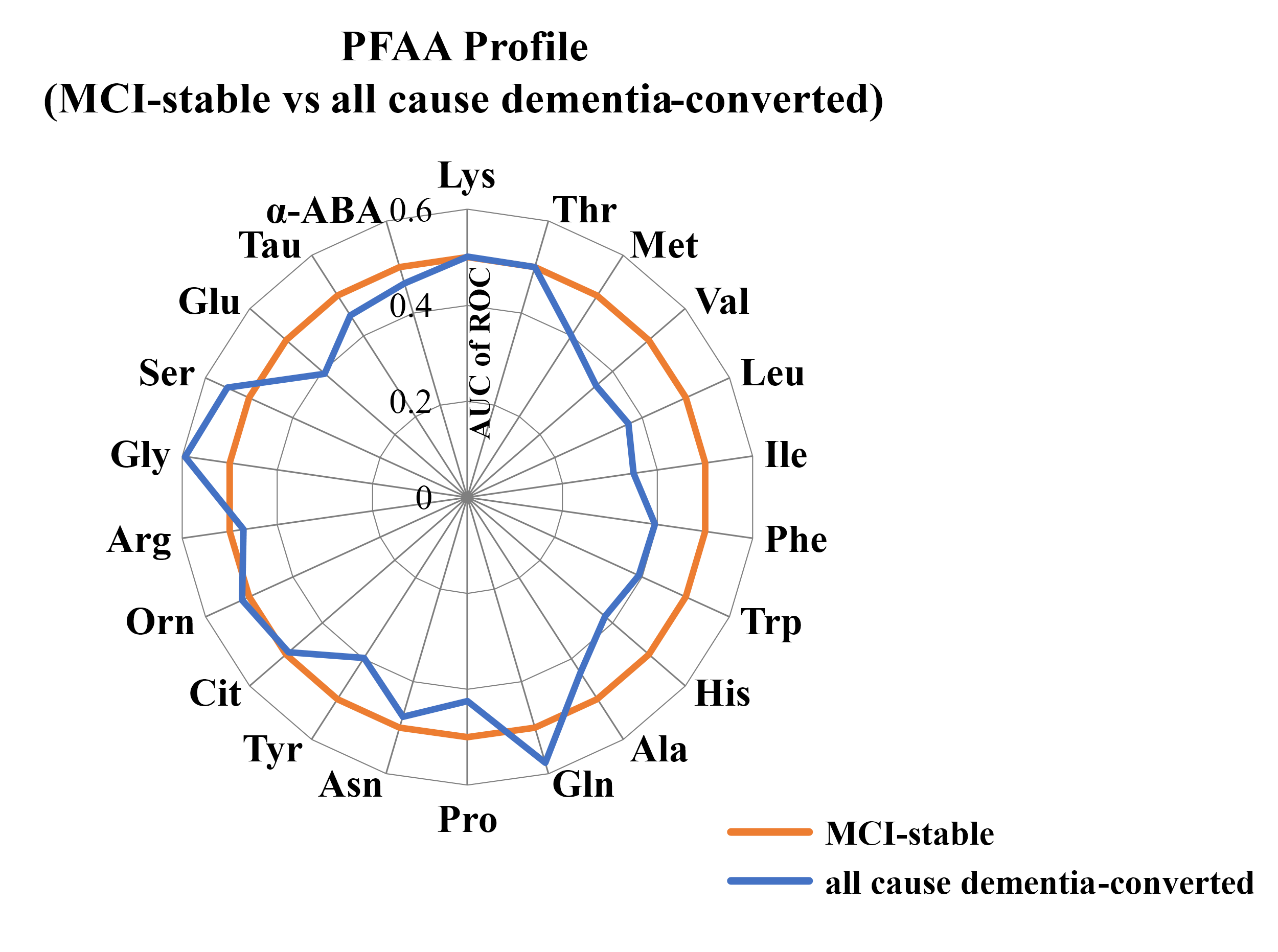


# **Supplementary Figure 1. Comparison of PFAA concentrations between MCI-stable and all cause dementia-converted individuals.** Differences in plasma free amino acid (PFAA) concentrations between groups are described by the ROC of AUC.


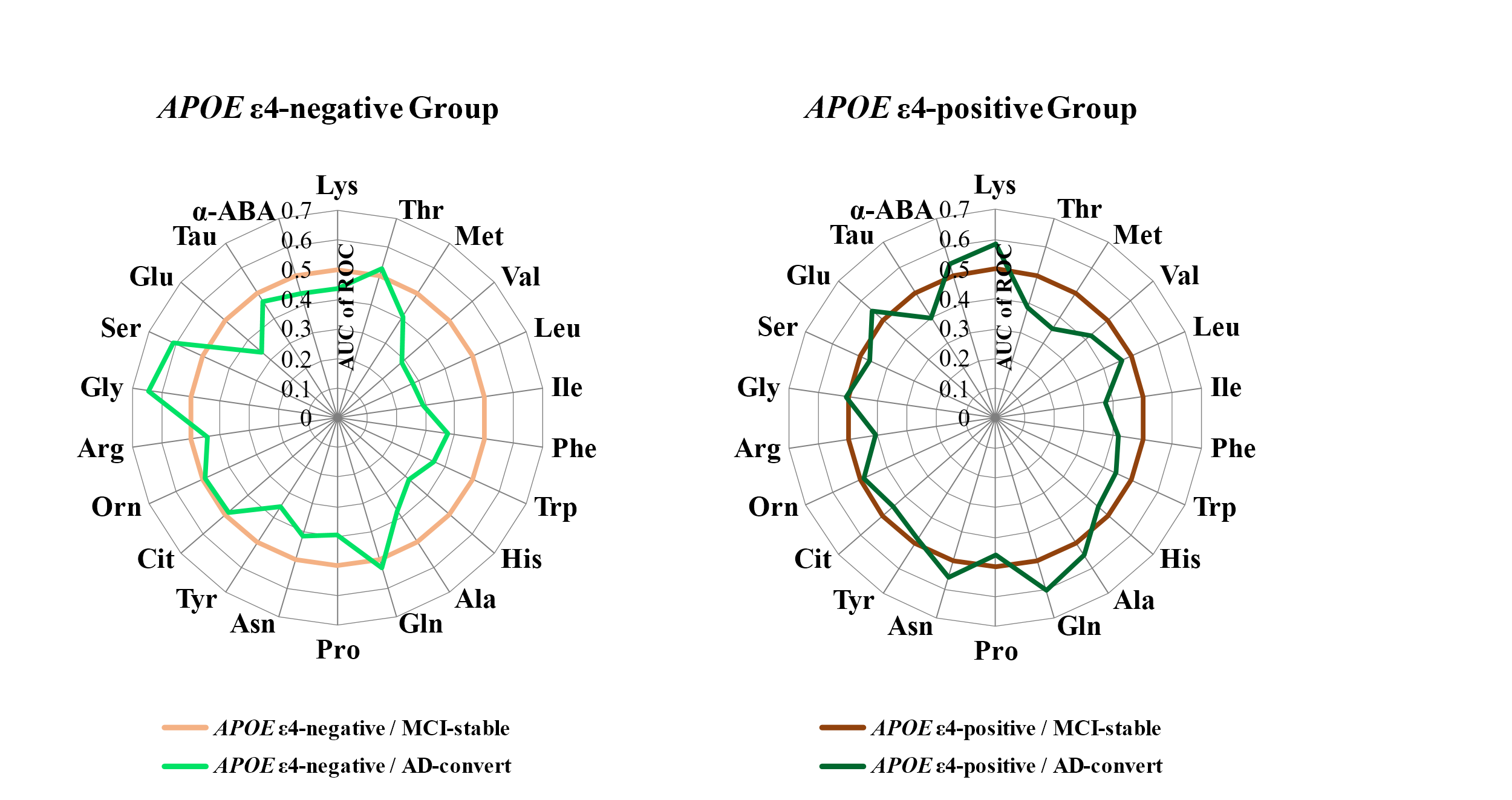


# **Supplementary Figure 2. Stratified analysis of MCI individuals by *APOE4* positive/negative status.** Differences in plasma free amino acid (PFAA) concentrations between diagnostic groups are described by the ROC of AUC. In each genetic group, the MCI-stable label was set as the control.


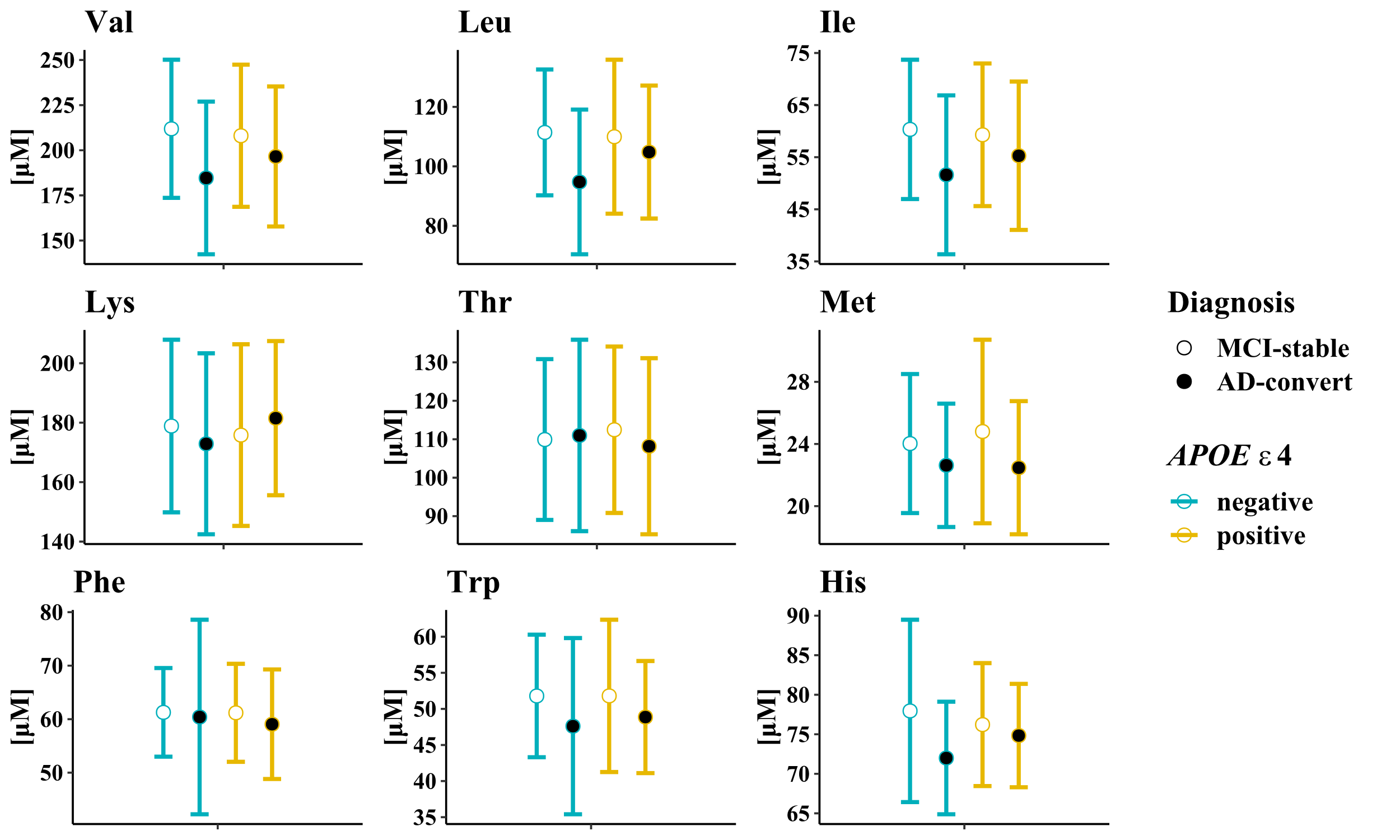


# **Supplementary Figure 3. Plasma EAA concentrations stratified by clinical diagnosis and APOE4 possession.** All data are represented as the mean ± SD. The open circle and filled circle represent MCI-stable and AD-convert, respectively. The blue line and yellow line represent APOE4-negative and APOE4-positive patients, respectively.
